# Recent Advances on Seaweed-Derived Pigments for FoodApplication and Current Legal Framework

**DOI:** 10.3390/foods14183265

**Published:** 2025-09-20

**Authors:** Elsa F. Vieira, Lígia Rebelo Gomes, Clara Grosso, Cristina Delerue-Matos

**Affiliations:** 1 REQUIMTE/LAQV, Instituto Superior de Engenharia do Porto, Instituto Politécnico do Porto, Rua Dr. António Bernardino de Almeida 431, 4249-015 Porto, Portugal; claragrosso@graq.isep.ipp.pt (C.G.); cmm@isep.ipp.pt (C.D.-M.); 2 FP-ENAS-Faculdade de Ciências de Saúde, Escola Superior de Saúde da UFP, Universidade Fernando Pessoa, Rua Carlos da Maia, 296, 4200-150 Porto, Portugal

**Keywords:** seaweed-derived pigments, chlorophylls, carotenoids, phycobiliproteins, legislation, food industry

## Abstract

The increasing demand for natural and health-promoting food ingredients has spotlighted seaweed-derived pigments as promising alternatives to synthetic colorants. This review explores the potential of chlorophylls, carotenoids, and phycobiliproteins extracted from various seaweed species for use in the food industry. These pigments offer not only a wide range of colors but also exhibit bioactivities such as antioxidant, anti-inflammatory, and anticancer effects. The paper discusses recent advancements in sustainable aquaculture practices, extraction, purification, and stabilization techniques, including green and microencapsulation methods, to enhance pigment yield and shelf life. Furthermore, it highlights the regulatory landscape in the European Union and the United States, identifying key differences and challenges regarding pigment approval and commercialization. Despite their potential, large-scale industrial adoption remains constrained by technical, economic, and regulatory hurdles. Bridging these gaps through optimized bioprocesses and safety assessments is essential to fully leverage seaweed pigments in food systems.

## 1. Introduction

The growing health and environmental concerns surrounding synthetic colorants have spurred a global shift toward natural alternatives in the food industry. Synthetic dyes, while cost-effective and stable, have been associated with adverse effects on human health (e.g., cancer, skin, and respiratory problems) and ecological systems (e.g.,reducingwater quality, killing aquatic life, and disrupting entire food webs), leading to increased consumer demand for clean-label products. In response, natural pigments, particularly those derived from marine sources such as seaweeds, are gaining momentum as promising substitutes. The global natural colorant market is projected to reach $3.2 billion by 2027, reflecting this paradigm shift toward safer and more sustainable food ingredients [1].

Among natural sources, seaweeds represent a rich and largely untapped reservoir of pigments with both functional and nutritional value [2]. Seaweeds are broadly classified into brown (Phaeophyceae), red (Rhodophyta), and green (Chlorophyta) family, and contain diverse pigments such as chlorophylls, carotenoids (e.g., β-carotene, xanthophylls), and phycobiliproteins [3]. Chlorophylls induce a green hue, carotenoids produce yellow-to-red shades, and phycobilin’s generate blue-to-red colors. These compounds not only provide a broad spectrum of colors for food applications but also exhibit notable beneficial therapeutic activities, including anticancer, antioxidant, anti-obesity, anti-inflammatory, neuroprotective, anti-angiogenic, anti-inflammatory, antidiabetic, and immunomodulatory effects [3,4]. Hence, seaweed-derived pigments offer dual functionality in the food industry, serving as both colorants and health-promoting agents, aligning with current trends in functional foods and nutraceuticals.

The seaweed industry has an annual value of USD $5.5–6 billion, with 90% of the production being for human food products, mainly for their high nutritional value [5]. However, despite their potential, the industrial exploitation of seaweed pigments remains limited in Western markets, primarily due to technical, economic, and regulatory challenges. Factors such as pigment instability, variability due to environmental conditions, and extraction complexity pose obstacles to their large-scale adoption. However, advances in bioprocessing technologies, including enzymatic extraction, ultrasound-assisted methods, and supercritical fluid extraction, have improved yield and pigment stability, bringing commercial-scale production closer to feasibility [3,4].

Importantly, the regulatory landscape surrounding the use of seaweed pigments in food products plays a pivotal role in determining their market potential. While several pigments like β-carotene and astaxanthin are already approved and widely used, others, such as fucoxanthin and phycobiliproteins, remain under regulatory scrutiny [6]. Regional differences in legislation, particularly between the European Union and the United States, further complicate the approval and commercialization process.

This review aims to provide a comprehensive overview of the status of seaweed-derived pigments in the food industry, with a particular focus on their production, functionality, and extraction methods. Special attention is given to the legal and regulatory frameworks governing their approval and use in Europe and the United States, highlighting the challenges and opportunities for integrating these marine-derived compounds into the global food colorant market.

## 2. Materials and Methodology

In April 2025, a non-systematic search was performed using online databases like Web of Science, SCOPUS, Science Direct, Springer, PubMed, MDPI, Wiley, and Google Scholar. The search used keywords such as “algae and seaweed,” “seaweed pigments,” “seaweeds-derived pigments and food application,” and “seaweeds-derived pigments and legislation.” Many studies published between 2009 and 2025 were found. However, only English-language articles focused on seaweed-derived pigments, their extraction methods, purification and stabilization, and use in food industry, were selected for review.

## 3. Seaweed-Derived Pigments

Seaweeds are taxonomically classified into three primary groups, each encompassing a broad diversity of species: brown seaweed (Phaeophyceae, Heterokontophyta; approximately 1750 species), red seaweed (Rhodophyta; ~6000 species), and green seaweed (Chlorophyta; ~1200 species) [7]. Several brown (e.g., *Himanthalia elongata*, *Undaria pinnatifida*, *Laminaria ochroleuca*, *Laminaria saccharina*, *Porphyra* spp., *Cystoseirabarbata*, *Sargassum fusiforme*, *Sargassum thunbergii*, *Fucus serratus*, *Saccharina japonica*, *Eisenia bicyclis*), green (e.g., *Caulerpa racemosa*, *Ulva Lactuca*, *Ulva rigida*, *Ulva flexuosa*), and red (*Kappaphycusalvarezii*, *Gracilaria verrucosa*, *Gracilariasalicorna*, *Gracilarialanceola*, *Gracilaria edulis*, *Gracilariacurticate*, *Gracilaria caudata*, *Gracilaria chilensis*, *Gracilariagracilis*, *Gelidiumpusillum*, *Laurencia catarinensis*, *Laurencia papilosa*, *Grateloupiaturuturu*, *Hypneaesperi*, *Asparagopsistaxiformis*, *Porphyridium purpureum*, *Porphyra* spp.) seaweed species have been recognized as rich sources of natural pigments [3,8,9]. Seaweed-derived pigments are primarily categorized into three major classes: carotenoids, chlorophylls, and phycobiliproteins.

**Figure 1:**
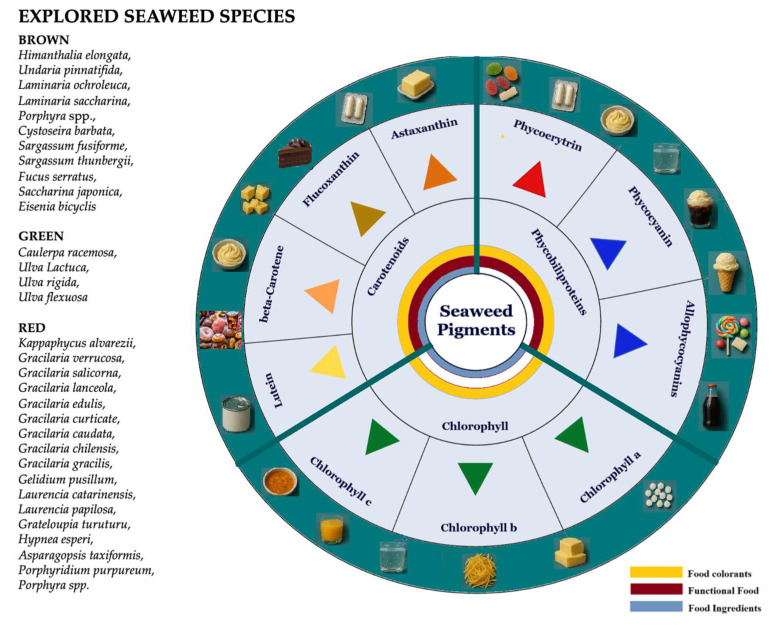
Food application as colorants of different types of seaweed-derived pigments.

The increasing interest in natural pigments, particularly those associated with health-promoting properties, has been largely driven by consumer demand for food additives that are non-synthetic, safe, and capable of providing a broad spectrum of colors. To address this demand, there is a growing need to develop optimized bioprocessing strategies for the efficient extraction, purification, and formulation of seaweed pigments into stable, application-ready forms. Figure 1 provides a comprehensive overview of the pigment diversity found in seaweeds, highlighting their potential applications as food colorants, as reviewed in this study.

### 3.1. Seaweeds Chlorophylls

**Figure 2:**
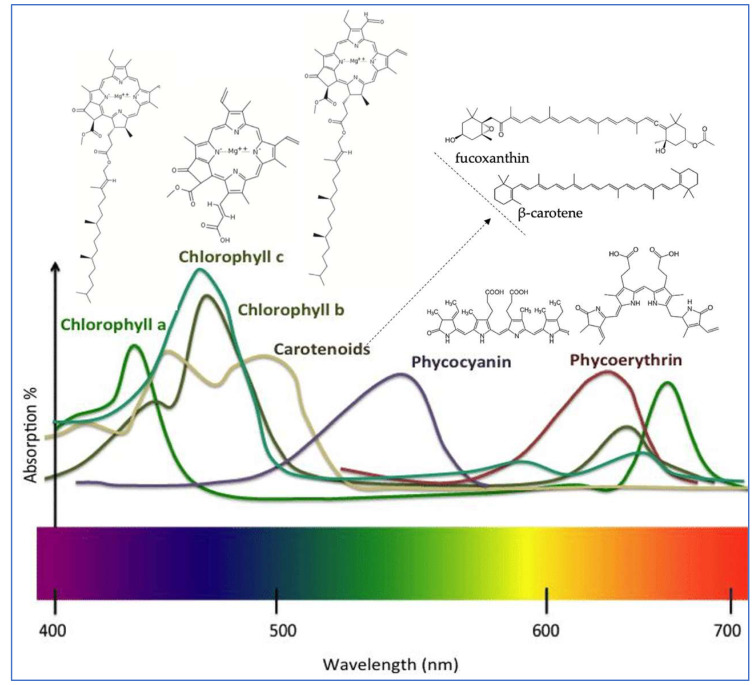
Chemical structures and spectrum of major pigments found in seaweeds.

Chlorophylls are key secondary metabolites found in seaweeds that are not only essential for photosynthesis but have also been associated with numerous biological functions in humans, including the enhancement of tissue regeneration, immune modulation, digestive health, and blood circulation [3]. Chlorophyll is made up of two main parts: a porphyrin ring, which helps it bind metals, and a long carbon chain called phytol, the common structure is presented in Figure 2. In seaweeds, there are four types of chlorophyll, with the most important being chlorophyll a, b, and c. Chlorophyll a is the most common in seaweeds. It has the formula C_55_H_72_MgN_4_O_5_ and appears blue–green, absorbing light mainly between 660 and 665 nm; chlorophyll b (C_55_H_70_MgN_4_O_6_) is found only in green seaweed. It is green–yellow and absorbs light between 642 and 652 nm, while chlorophyll c is found in brown seaweeds and has a blue–green color, absorbing between 447 and 452 nm [10]. Among seaweeds, Chlorophyceae species are the most extensively studied for their chlorophyll content. *Enteromorpha prolifera* has demonstrated potent antioxidant activity, attributed to its high pheophorbide a content, presenting DPPH radical-scavenging, reducing power, and hydroxyl radical-scavenging activities [11]. Additionally, chlorophyll a, from several brown seaweed species, has shown notable peroxyl radical-scavenging capacity, which may be further enhanced by synergistic interactions with vitamin E [12].

The global annual production of chlorophyll-based additives is estimated to approach 1.2 billion units [13]. Chlorophylls and their copper derivatives, such as chlorophyllin, are widely utilized as natural food colorants and are designated by E-numbers E140 and E141, respectively. These pigments are marketed according to their solubility properties: E140i (chlorophyll) is lipophilic, while E140ii (chlorophyllin’s) refers to the water-soluble form, typically sold as a powder, obtained by saponification of the vegetable extract [2]. However, both forms are chemically unstable, with the green color tending to shift rapidly to brown under processing or storage conditions [14]. For this reason, the food industry commonly prefers E141, a more stable colorant produced by introducing copper into the respective chlorophyll solutions, whether lipophilic or hydrophilic. Copper chelation stabilizes the chlorophyll molecule, effectively preserving the green hue regardless of processing conditions or shelf life duration. In summary, E141 consists of copper complexes of chlorophyll derivatives. E141i (copper chlorophylls) refers to the oil-soluble variant, while E141ii (copper chlorophyllin’s) denotes the water-soluble form [15].

These compounds are considered safe and are used for their antioxidant and color-enhancing properties in a variety of food products, including pasta, flavored vegetable oils, ice cream, fruit jellies, fruit juices, beverages, candies, canned peas, and soups [3,16,17]. Practical applications of seaweed-derived chlorophylls in food systems have demonstrated both functional and sensory benefits. For example, the incorporation of chlorophyll-rich seaweed extracts into jelly desserts significantly enhanced their visual appeal, with color stability retained over one month at ambient temperature [18]. Similarly, the addition of *Caulerpa racemosa* powder to semi-sweet biscuits resulted in decreased yellowness, redness, and lightness values due to chlorophyll pigments. However, higher seaweed concentrations correlated with a reduction in sensory acceptability, indicating potential limitations in application at elevated doses [19]. In baked goods, chlorophyll-rich seaweed powders have been used to develop functional breads, which exhibit a shift in crumb and crust coloration toward darker shades with green–blue hues, reflecting high pigment concentrations [20]. Chlorophyllin, a water-soluble semi-synthetic derivative containing copper, is widely used in commercial food products such as candies, ice cream, desserts, and cookies, owing to its enhanced thermal and oxidative stability [18,19,21]. Beyond coloration, chlorophyllin has been shown to reduce microbial load, thereby contributing to improved food safety and shelf life [22]. Furthermore, chlorophyll and its derivatives can influence the texture of food products. When encapsulated or co-processed with macronutrients such as lipids, proteins, or starches, chlorophyll-based pigments may modify creaminess, firmness, or viscosity, adding functional value beyond their visual appeal [23]. Chemical structures of major pigments found in seaweeds.

### 3.2. Seaweeds Carotenoids

Carotenoids are a diverse group of naturally occurring tetraterpenoid yellow, green, and orange pigments, widely distributed among seaweeds, playing essential roles in photosynthesis and photoprotection. Carotenoids absorb light energy for chlorophyll and protect cells from oxidative damage induced by ultraviolet (UV) radiation through their potent antioxidant properties [4]. Over 600 carotenoids have been identified, to date [24], and they are broadly classified into two major categories based on their molecular structure: the pure hydrocarbon (carotenes, e.g., a-carotene, β-carotene, and lycopene) and the oxygenated (alcoholic) derivatives (xanthophylls, e.g., lutein and zeaxanthin), Figure 2.

Carotenes are hydrocarbon carotenoids that lack oxygen atoms. Examples found in seaweeds include α-carotene, β-carotene, γ-carotene, lycopene, phytoene, and phytofluene. Some of these, such as α-carotene, β-carotene, γ-carotene, and β-cryptoxanthin, possess a β-ionone ring, enabling them to function as provitamin A compounds in humans. In contrast, lycopene, lutein, and zeaxanthin lack provitamin A activity but still exhibit notable antioxidant potential [4]. Xanthophylls, which are oxygenated derivatives of carotenes, represent the dominant class of carotenoids in brown seaweed and is largely responsible for their brownish coloration. This class of carotenoids includes fucoxanthin, diadinoxanthin, diatoxanthin, lutein, zeaxanthin, neoxanthin, astaxanthin, siphonaxanthin, vaucheriaxanthin, and loroxanthin. Among these, fucoxanthin is the principal carotenoid in brown seaweeds, playing a vital role in light absorption within the blue–green spectrum and contributing significantly to photoprotection under high light stress. *Undaria pinnatifida* contains the highest level of fucoxanthin among several seaweed species [3]. This carotenoid has gained attention for its multifunctional bioactivity, including antioxidant, anti-inflammatory, anticancer, antidiabetic, anti-obesity, neuroprotective, and photoprotective effects [25]. The incorporation of seaweed carotenoids in food products has been extensively studied. For instance, β-Carotene has been incorporated in a wide range of food products, such as cheese, yogurts [26,27], gummy, candies [28], puddings [27], among others. Fucoxanthin extracted from *Cystoseirabarbata* has been shown to enhance the oxidative stability, color retention, and shelf life of cured turkey meat sausages [29]. Other food applications include baked products [30],in ground chicken breast meat [31], or shrimp paste [32].

### 3.3. Seaweeds Phycobiliproteins

Phycobiliproteins are a class of intensely colored, water-soluble pigment–protein complexes embedded within phycobilisomes, where they efficiently absorb and transfer light energy to chlorophyll a in the photosystems [33]. Phycobiliproteins are composed of α and β polypeptide subunits (~30–35 kDa) that are covalently bound to linear tetrapyrrole chromophores known as phycobilins. Phycobiliproteins are generally classified based on their absorption maximum (Amax) in the visible region of the spectrum. The most common phycobiliproteins are phycocyanin (A_max_= 610–625 nm, blue), allophycocyanin (A_max_ = 650–660 nm, blue–green) and phycoerythrin (A_max_ = 490–570 nm, red) [34]. Their structure is shown in Figure 2. Beyond their photobiological role, phycobiliproteins have attracted increasing attention due to their diverse biological activities, including, antioxidant, anti-inflammatory, anticancer, antimicrobial, neuroprotective, and antimetabolic (e.g., antidiabetic, anti-obesity). These bioactivities make these pigments valuable candidates for applications in biomedicine, pharmaceuticals, nutraceuticals, and functional foods [33].

Phycobiliproteins have been widely explored as natural food colorants due to their bright pigmentation, fluorescent properties, and non-toxic, non-carcinogenic nature. Phycoerythrin is a natural protein pigment found in the red alga *Kappaphycusalvarezii*. Other red seaweed species, namely *Gracilaria caudata*, *Laurencia catarinensis*, and *Porphyridium purpureum*, have been studied for optimized phycobiliproteins production. This pigment displays a vibrant pink color at physiological pH, and changes in pH produce a wide range of attractive pigments for use in cosmetics and functional foods products [35]. Phycoerythrin has been used as a substitute for synthetic colorants to increase the color of food, correct natural deviations in color and improve product quality. In the food industry, they can be found in chewing gums, ice creams, jellies, health drinks, and sweeteners [35,36].

## 4. Novel Extraction and Purification Methods for Seaweed-Derived Pigments

### 4.1. Extraction Strategies

Although solvent extraction method is straightforward and very efficient for pigments extraction from seaweeds, it has some major drawbacks [4]. Most of the solvents used for pigment extraction (e.g., methanol, n-hexane, dichloromethane, diethyl ether, and acetone) have safety concerns. For instance, chloroform is classified as extremely hazardous while hexane may cause damage to organs through prolonged or repeated exposure. In addition, high solvent consumption is involved, and several concentration and clean-up steps are required [3,4]. Alongside the health-focused shift, environmental awareness is also growing, leading to the emergence of green extraction techniques and green analytical chemistry. These methods aim for safer, more sustainable, and affordable procedures that minimize solvents, energy, and hazardous substances, offering economic and environmental benefits to avoid the limitations and disadvantages of conventional techniques [37]. Newer methodologies like supercritical fluid extraction (SFE), ultrasound-assisted extraction (UAE), pressurized liquid extraction (PLE), and microwave-assisted extraction (MAE) are currently in use for the extraction of seaweed-derived pigments [3].

**Figure 3:**
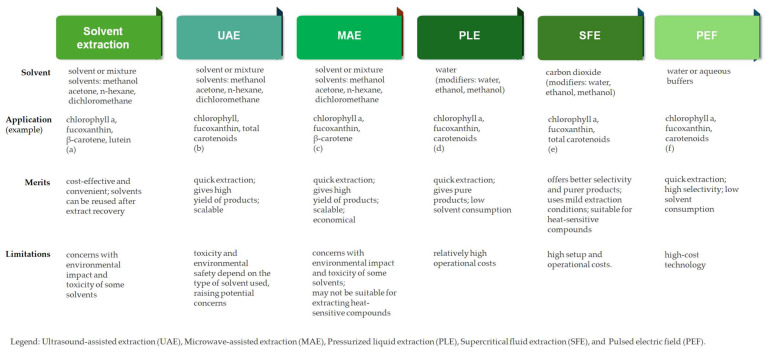
Extraction techniques of seaweed-derived pigments. (a) Amorim et al., 2012 [38]; (b) Zhu et al., 2012 [39]; (c) Lopes et al., 2024 [40]; (d)Zanng et al., 2025 [41]; (e) Saravana et al., 2017 [42]; (f) Castejón et al., 2021 [43].

Overall, maximizing pigment yield requires careful optimization of factors such as the extraction solvent, solvent-to-sample ratio, extraction time, temperature, pH, and particle size. Additionally, the choice of biomass form, whether fresh, spray-dried, freeze-dried, or oven-dried, plays a crucial role in selecting the most effective extraction method [37]. The recent progress in natural seaweed pigments extraction was recently reviewed by [3]. Figure 3 gives an overview of the advantages and drawbacksof these, along with examples of applicability for extraction of seaweed pigments [38,39,40,41,42,43].

Ultrasound-assisted extraction (UAE) is a fast and solvent-efficient method that can yield high amounts of pigments. However, temperature increases during the process must be controlled to avoid damaging the pigments. While UAE is suitable for large-scale extraction from seaweeds, it generally produces extracts with lower purity [3]. For instance, it was effectively used to extract chlorophylls from the brown seaweed *Undaria pinnatifida*, reaching a combined maximum yield of 0.54 mg/mL for chlorophyll a and b [39]. Pereira et al. (2020) [44] optimized UAE for extracting phycobiliproteins from the red seaweed *Gracilariagracilis.* Compared to traditional maceration, UAE (at 45 kHz, 400 W, for 7.5 min) resulted in lower yields, between 1.48 and 1.99 mg/g. UAE was also tested alone and alongside methods like maceration, liquid nitrogen treatment, homogenization, and freeze–thawing to extract water-soluble pigments such as R-phycoerythrin (R-PE) and R-phycocyanin (R-PC) from *Gelidiumpusillum*. Mittal et al. (2020) [45] reported that combining maceration with UAE achieved the highest efficiency—77% for R-PE and 93% for R-PC.

Other environmentally friendly techniques include pulsed electric field-assisted extraction (PEF), microwave-assisted extraction (MAE), and supercritical CO_2_ extraction (SFE). However, high temperatures can degrade pigments, and PEF may cause electrode corrosion or metal contamination, limiting their application [4]. Despite these limitations, some promising results have been reported. Poojary et al. (2016) [46] used pressurized liquid extraction (PLE) on *Himanthalia elongata*, identifying fucoxanthin and zeaxanthin as major compounds (0.82 mg/g and 0.13 mg/g, respectively). Quitain et al. (2013) [47] extracted fucoxanthin from *Undaria pinnatifida* using SFE, recovering 80% at 40 °C for 180 min. Honda et al. (2022) [48] also used SFE (30 MPa, 160 °C, 30 min) with ethanol as a co-solvent, achieving a 29.5% fucoxanthin recovery. Similarly, Saravana et al. (2017) [42] found that sunflower oil as a co-solvent improved pigment extraction from Saccharina japonica more effectively than other oils or ethanol. Optimal SFE conditions (50 °C, 300 bar, 2% sunflower oil) yielded 2.391 mg/g of total carotenoids and 1.421 mg/g of fucoxanthin. Although techniques like UAE, MAE, PLE, and SFE can produce high-purity pigments suitable for food use, their high cost and technical complexity limit their industrial scalability.

### 4.2. Purification Strategies

The purification of seaweed-derived pigments involves a diverse array of techniques tailored to the chemical nature of the target compound. Advances in green extraction technologies and scalable chromatographic systems have significantly improved the efficiency and sustainability of these processes [34]. In the case of chlorophylls, liquid–liquid partitioning, commonly employing hexane–water systems, is used to separate from non-pigment impurities. Further purification is achieved using column chromatography with silica gel or Sephadex resins [49]. Thin-layer chromatography (TLC) can serve as both a qualitative assessment and a minor purification technique [50]. Carotenoids are commonly subjected to saponification to remove lipids and chlorophylls. Silica gel or C18 column chromatography is used for separation, and high-performance liquid chromatography (HPLC), especially reverse-phase, remains the gold standard for achieving high-purity compounds [34]. Purification of phycobiliproteins typically begins with ammonium sulfate precipitation (or salting out) to concentrate the proteins. Further refinement involves ion-exchange chromatography (e.g., DEAE-cellulose or Q-Sepharose) and size-exclusion chromatography (gel filtration). Ultrafiltration and dialysis are commonly employed to remove low-molecular-weight impurities and concentrate the final product [51]. This methodology is interesting because it is fundamentally “green”, cost-effective, environmentally friendly, and suitable to be used on an industrial scale [34].

## 5. Stabilization Techniques to Prevent Seaweed-Derived Pigments Degradation

Seaweed-derived pigments are very sensitive to environmental and processing conditions, limiting their stability and shelf life in food applications. Also, the composition and physicochemical properties of the food matrix in which they are incorporated highly influence the stability of seaweed-derived pigments. The influence of the food matrix is evident across diverse product categories, as illustrated in Table 1. To enhance pigment stability in complex food systems, several technological strategies have been developed, including encapsulation techniques (spray drying, freeze drying, liposomes, nanoemulsions), interfacial engineering of emulsions with protein–polysaccharide multilayers, antioxidant supplementation, and careful regulation of pH, oxygen, and light exposure [52]. For example, in acidic beverages, chlorophylls rapidly undergo pheophytinization [53], whereas carotenoids encapsulated in nanoemulsions or cyclodextrin inclusion complexes demonstrate improved resistance to degradation [54]. In dairy emulsions, caseins and whey proteins not only stabilize carotenoids but also enhance their bioaccessibility after digestion [55]. In bakery systems, microencapsulation with maltodextrins, gums, or proteins can substantially reduce pigment degradation [56]. Despite these advances, significant challenges remain in unlocking the complete functional and technological potential of seaweed-derived pigments as natural colorants across food matrices.

The instability of chlorophylls under environmental and processing conditions, such as light, oxygen, pH, heat, and packaging, presents a major challenge to their broader use in food systems. To enhance pigment stability and maintain functionality, several encapsulation techniques have been explored. Nanoemulsified chlorophyll formulations have demonstrated improved pigment stability and bioavailability [58]. Likewise, carotenoids, such as β-carotene and fucoxanthin, suffer from poor thermal and acidic stability due to its polyunsaturated structure and are highly vulnerable to degradation under light, heat, oxygen, and humidity. This instability, combined with its low bioavailability, restricts its incorporation into food products, making it necessary to adopt strategies such as microencapsulation [28,59]. Regarding phycobiliproteins, its potential as food colorant is significantly dependent on external factors like light, temperature, and pH fluctuations, which can lead to protein denaturation and color degradation [35]. To overcome these difficulties, several techniques could be used, such as the addition of food stabilizers, protein structural transformation, and microencapsulation. For instance, benzoic acid, ascorbic acid, citric acid, NaCl_2_, CaCl_2_, NaN_3_, dithiothreitol, and sucrose have been used to protect phycobiliproteins denaturation, but some chemical preservatives might be harmful and inadmissible in some food application. Therefore, encapsulation has been the safest technique for thermally unstable phycobiliproteins [35].

Overall, to overcome low stability of seaweed-derived pigments, encapsulation techniques are being developed to protect seaweed-derived pigments from adverse conditions, enhance their stability, and improve their delivery and performance in end-use applications. Encapsulation can shield pigments from moisture, light, temperature extremes, and oxidative degradation, thus extending their usability in the food, pharmaceutical, and cosmetic industries. The micro-sized particles are covered by wall material or encapsulant agent, which protects and isolates these particles from ambient conditions. The choice of the solvent medium varies with the solubility of core material and encapsulants. The properties of these coatings or matrices decide the release kinetics of bioactive or functional ingredients under specific conditions [60].

There are three categories of microencapsulation: physical, chemical, and physicochemical. Physical microencapsulation is based on physical and mechanical principles, and the formation of the shell depends on solid–liquid phase transition under heating or solubility reduction due to solvent evaporation. Spray drying, spray cooling, air suspension, envelop–combination, extrusion, supercritical solution, porous centrifugal, electrostatic binding, solvent evaporation, and rotary separation belong to this category [61]. Chemical methods are based on chemical reactions, in which the monomers polymerize to form the polymer shell. Examples of this category include interfacial polymerization, in situ polymerization, and piercing-solidification. In physicochemical processes, the pre-dissolved shell-forming materials precipitate from the solution following the variation in temperature, pH value, or electrolyte concentration, and gradually deposit on the surface of the core material to form the shell. Simple coacervation, complex coacervation, phase separation, drying bath, powder bed grinding, melting–dispersion–condensation, and capsule–core exchange are some of these methods [61]. Recent review papers have thoroughly compiled various microencapsulation techniques, detailing the processes underlying each method [62,63,64]. Table 2 presents several studies performed on the micro- and nanoencapsulation of seaweed-based pigments using different techniques, mainly physical ones.

### 5.1. Stabilization of Chlorophylls

Chlorophylls are stable green pigments in natural sources. However, during food processing and storage, especially when the temperature or pH changes, they can break down and change color. The most common change is the loss of the magnesium ion (Mg^2+^) in the pigment, which makes the bright green color turn into a dull brown, forming compounds called pheophytins and pheophorbides. This color change is a problem for the food industry, as consumers often see brown or faded green vegetables as low quality. To prevent this, different methods have been developed to keep the green color during processing. One early method involvedadding alkaline substances to stop the color change, but this sometimes made the vegetables too soft or gave them an unpleasant taste, so it was not widely used. A better solution was to replace the lost magnesium with zinc or copper ions, forming more stable green pigments called metallochlorophylls. This process, known as “re-greening”, helps vegetables look fresh and green again. A well-known example is the “Veri-green” method, where vegetables are cooked in saltwater with zinc or copper to restore the color. However, this method has regulatory limits—especially with zinc—since the FDA only allows a small amount (75 ppm), which may not be enough to achieve the desired green color. To address this, newer techniques are being developed. These include encapsulating chlorophyll with protective materials like gum Arabic, maltodextrin, modified starch, or whey proteins. These approaches help protect the color without needing high levels of metal salts.

Dewi and Purnamayati (2023) [17] evaluated the effects of *Caulerpa* sp. microcapsules, on the physical, chemical, and sensory properties of seaweed-based jelly drinks. Microencapsulated chlorophyll extracted from *Caulerpa* sp. (chlorophyll content = 0.07−0.10 ppm) was incorporated into the jelly drinks at concentrations ranging from 0 to 4000 ppm. The addition of the microcapsules influenced the drinks’ appearance, with the color turning progressively darker green as the concentration increased. Higher concentrations also led to increased viscosity and elevated levels of dissolved solids, as well as increased total sugar content, dietary fiber, and chlorophyll levels. Conversely, as the concentration of *Caulerpa* sp. increased, syneresis decreased. Total phenolic content (TPC) and antiradical activity (against DPPH^•^) also rose with higher microcapsule concentrations. While the chemical characteristics were enhanced at higher concentrations, sensory evaluation showed that panelists preferred jelly drinks with a lighter color and lower *Caulerpa* sp. microcapsules content.

Another study investigated the stability of encapsulated pigments extracted from *Sargassum* sp. using 100% acetone. The pigments were encapsulated via freezedrying, using maltodextrin and Tween-80 as encapsulating agents, resulting in a yellowish-green pigment powder [65]. The main chlorophyll derivatives found in the microcapsules were chlorophyll c and pheophytin a, while trans-fucoxanthin was the dominant carotenoid (107.2 µg/g), followed by cis-fucoxanthin, zeaxanthin, and E-carotene. To assess pigment stability, the freeze-dried powder was stored in the dark at temperatures of 28, 45, and 65 °C. HPLC results showed that the shoulder peak of fucoxanthin at 447 nm degraded more rapidly at higher temperatures than the Soret and Qy bands of pheophytin a, indicating greater thermal sensitivity. Pigment degradation was also reflected in color changes: lightness (L)* increased over time, while redness (a)* and yellowness (b)* values decreased. The b* value was suggested to represent the presence of pheophytin a and chlorophyll c, whereas a* was associated with thermolabile carotenoids. The study further estimated the half-life of the encapsulated pigments. At 28 °C, the half-life was approximately 63 days, which could be extended to 118 days when stored at 21 °C, indicating improved pigment stability at lower temperatures.

Ledari et al. (2024) [66] also encapsulated chlorophylls, which were extracted with 90% acetone from *Ulva intestinalis*. The chosen encapsulated agents were maltodextrin and whey protein isolate. They tested two drying methods, namely spray drying and freeze drying. The optimum combination of wall and core materials to achieve the highest encapsulation efficiency was obtained by central composite design. The microcapsules produced by freeze drying had higher antioxidant activity (79.1% of DPPH^•^inhibition), higher encapsulation efficiency (91.2%) and chlorophyll content (89.67 μg/mL) than the microcapsules produced by the spray dryer. On the other hand, the highest solubility and the lowest moisture content were observed for microcapsules obtained through spray drying. Since the moisture content is one of the factors which could affect the shelf life of dried products, spraydrying seems to be the most promising technique in this study.

### 5.2. Stabilization of Carotenoids

Several encapsulation studies have been conducted with carotenoids, mainly with fucoxantin and β- carotene. Sun et al. (2018) [59] extracted fucoxanthin from *Undaria pinnatifida* with 80% ethanol, followed by 95% (*v*/*v*) methanol/n-hexane. Different biopolymers were tested for fucoxanthin encapsulation, namely hydroxypropyl-β-cyclodextrin, maltodextrin, isolated pea protein, whey protein isolate, gum Arabic, and gelatin. All the microspheres prepared had a spherical shape with a rough surface and sizes between 6.55 µm (using maltodextrin) and 9.26 µm (using gelatin). The encapsulation efficiencies ranged from 86.48% (for gum arabic microcapsules) to 97.06% (hydroxypropyl-β-cyclodextrin microcapsules). FTIR analysis showed that it was a physical encapsulation process, and no new chemical bonds were formed between the pigment and the biopolymers. The moisture content was between 2.68% and4.90%, with maltodextrin microcapsules showing the lowest content as well as the lowest water activity. A heat stability test showed that from all the encapsulated agents, maltodextrin, gum Arabic, and whey protein isolate improved the thermal stability of fucoxanthin. The degradation kinetics of the loaded fucoxanthin encapsulated with biopolymers also indicated that gum arabic and maltodextrin had a better protective effect on fucoxanthin. Results from the simulated digestion test in vitro also suggested that maltodextrin, gum Arabic, and whey protein isolate effectively protected fucoxanthin in the gastric acid environment and increased the release rate of fucoxanthin in the intestinal tract.

Wang et al. (2017) [73] isolated fucoxanthin from *Sargassum thunbergii* and synthetized microcapsules composed of palm stearin solid lipid core and a fish gelatin–gum arabic complex coacervate shell. The content of fucoxanthin extracted from this seaweed was 1.5 ± 0.2 mg/g dw. The microcapsules had smooth surfaces and 19.19 µm mean diameter. Encapsulation efficiency and loading capacity of microcapsules with fucoxanthin were 98.3% and 0.04%, respectively. The effect of temperature (4, 25 and 50 °C), relative humidity (33% RH and 80% RH), and dark/light on fucoxanthin stability was studied, showing that fucoxanthin in microcapsules presented higher stability than free fucoxanthin against these three parameters. The release of fucoxanthin from the microcapsules was investigated during the successive incubations in simulated gastrointestinal condition at 37 °C in the dark. The cumulative amount of fucoxanthin released from microcapsules was 22.92% and 56.55% in simulated gastric fluid and in simulated intestinal fluid, respectively.

Constantino and Garcia-Rojas (2023) [28] microencapsulated β-carotene by complex coacervation of amaranth carboxymethyl starch and lactoferrin for application in gummy candies. The results indicated that high affinity electrostatic attraction between CMS and LF can occur at pHs between 3.5 and 5.5 for complex coacervates formation. About 98% of β-carotene was microencapsulated in amaranth carboxymethyl starch and lactoferrin complex coacervates, at pH = 5. The microcapsules (4247.0 nm) presented well-defined spherical structure. Stability under high temperatures (50 °C) or UV radiation was tested, revealing good photolytic and thermal stabilities. Finally, the authors tested the simulation of the in vitro gastrointestinal digestion of β-carotene microcapsules and the release kinetics of microencapsulated β-carotene in food matrices. The digestion in the oral and gastric phases showed less than 10% β-carotene release rate, but about 70% of β-carotene was released in the intestines. In food matrices, 86% release rate was observed in soybean oil, and 19% in 50% ethanol. Finally, gummy candies were produced with and without β-carotene microcapsules. Regarding the texture, the hardness, gumminess, and chewiness were reduced after the addition of the microcapsules, whereas the adhesiveness, elasticity, and resilience were not affected. The β-carotene bioaccessibility after the in vitro simulation of the gastrointestinal digestion of the microcapsules was 27.92%, while the microcapsules added to the gummy candies were 22.44%. This shows that β-carotene microencapsulated was protected during the in vitro gastrointestinal digestion. The differences in the bioaccessibility of the pure microcapsule and those added in the gummy candies can be explained by several factors, including the complexity that a food matrix can present, making it difficult to properly digest the microcapsules incorporated in them.

Moraes et al. (2013) [72] produced powders containing β-carotene, hydrogenated phosphatidylcholine, and sucrose and characterized them in terms of crystallinity, morphology, thermal behavior, density, solubility, and hygroscopicity. The SEM micrographics showed perfectly spherical particles with no ruptures, diameters <3 µm in most cases, and the absence of agglomerates, crystals of β-carotene, and sucrose. The X-ray diffractogram of the proliposome confirmed homogeneity as it did not indicate any peaks of the pure sucrose or pure β-carotene, but there was one peak at 21.4° with major characteristics of the phospholipid. The proliposomes were also highly water-dispersible, which would facilize significantly the formation of liposomes at the hydration step. Concerning shelf life, the powder samples were stored under two different conditions, normal atmosphere and under vacuum. There were nearly no losses of β-carotene after 60 days of storage under vacuum, while storing the proliposomes under normal atmosphere led to a loss of 30% of the initial mass of β-carotene. Liposomes were produced by hydration of the proliposomes and a thickener agent was also added, xanthan gum. The liposomes produced (average diameter = 1500 nm; PDI ≈0.40; zeta potential ≈−40 mV) were stable over 3 months. The addition of xanthan gum also allowed the preservation ofthe incorporated β-carotene throughout the storage time. Only after100 days, approximately 25% of the initial mass of β-carotene was degraded.

Donhowe et al. (2014) [27] determined the effect of the microencapsulation method on the physical properties and in vitro release and bioavailability of three types of β-carotenes: a spray-dried powder of β-carotene and maltodextrin, a commercially available water-dispersible β-carotene powder, and chitosan coated β-carotene alginate. In vitro digestion trials were conducted with and without food matrices (yogurt, pudding) to elucidate the effect of food matrix on in vitro release and bioavailability. Microencapsulation method significantly affected water activity, moisture content, and particle size. Water activity was between 0.195 (chitosan–alginate beads) and 0.279 (water-dispersible powder); the moisture content ranged from 3.5% (maltodextrin powder) and 16.5% (chitosan–alginate beads); and particle size was between 10.5 µm (maltodextrin powder) and 942.8 µm (chitosan–alginate beads). Regarding β-carotene release, water-dispersible β-carotene and chitosan–alginate beads achieved the highest and the lowest release rate (93% and 7.7%), respectively. Digestion with a food matrix significantly decreased the release and bioavailability of the three β-carotene types, with pudding superior to yogurt in terms of release and bioavailability, except for maltodextrin powder.

β-Carotene has also been microencapsulated using more advanced techniques, such as supercritical micronization [57,59,60]. Paz et al. (2012) [69] studied the formulation of β-carotene with poly-(ε-caprolactone) by the Particles from Gas Saturated Solutions (PGSS) process. Particle sizes in the range of 270–650 μm with a β-carotene content of up to 340 ppm were obtained using polycaprolactone with a molecular weight of 10,000 g mol^−1^, while using a polycaprolactone with a molecular weight of 4000 g mol^−1^, the particle size was reduced to 110–130 μm. The influence of several process parameters on particle size and β-carotene content was studied, including pressure (11 and 15 MPa), temperature (50 and 70 °C), time of contact between CO_2_ and polymer melt for mixture homogenization (60, 120 and 240 °C), and molar ratio β-carotene/polymer (0.13, 0.16 and 0.25). Regarding the molar ratio, when it was increased, the particle size increased as well together with the β-carotene content. Bigger particles were obtained when the pressure and temperature inside the mixing chamber were 15 MPa and 50 °C, respectively, compared to the results obtained at 70 °C and 11 MPa. The highest β-carotene concentrations (306–336 ppm) were obtained at high pressures and temperatures (70 °C and 15 MPa, respectively) and with short homogenization times (60 min). The same research group also used PGSS to encapsulate β-carotene with soybean lecithin. The influence of the main process parameters (pressure, temperature, gas-to-product ratio, and concentration of the carrier material) was carried out. Dry particles of 10–500 μm, constituted by fused spherical particles of less than 10 μm, have been obtained, with β-carotene encapsulation efficiencies up to 60%. The best encapsulation efficiency (58.7%) was achieved using 114 °C, 10.1 MPa, 32 gas-to-product ratio and 62 g/L of soybean lecithin [64]. In another study, the solution enhanced dispersion by supercritical fluids (SEDS) technique was employed to encapsulate β-carotene in poly(hydroxybutirate-co-hydroxyvalerate) (PHBV) with dichloromethane as the organic solvent and CO_2_ as the antisolvent. The highest encapsulation efficiency (55.54%) was achieved using the following conditions: 30.11 mg/mL of β-carotene in solution, 30.70 mg/mL of PHBV in solution, temperature at 40 °C, pressure at 80 bar, solution flow rate at 1 mL/min, and antisolvent flow rate at 40 mL/min [67].

Few studies have been conducted with other carotenoids. Xia et al. (2012) [68] and Nerome et al. (2013) [71] encapsulated lutein and lycopene by supercritical antisolvent (SAS) and SEDS techniques, respectively. Proliposomes composed of lutein and hydrogenated phosphatidylcholine were prepared using SAS. Different conditions of pressure (8, 10, 12, 14, 16 MPa), temperature (35, 40, 45, 50, 55 °C), Lutein/hydrogenated phosphatydylcholine ratio (10, wt.%), and flow rate of liquid (0.5, 1.0, 1.5 mL/min) were investigated. At the optimum conditions tested (35 °C, 8 MPa, and solution flow rate of 1 mL/min), the lutein loading of the proliposomes reached 55 mg/g. SEM images showed that proliposomes were spherical and their size was around 200 nm with a narrow size distribution, which is good for forming the lutein liposomes later. XRD and DCS analysis showed that lutein is embedded completely in the hydrated phosphatydylcholine. When proliposomes was hydrated, the lutein liposome suspensions were formed automatically with an encapsulation efficiency of more than 90% after hydrating proliposomes. TEM images showed that these liposomes were larger than their parenteproliposomes (500 nm) and while liposomes were monolayer vesicle structures, proliposomes were solid spheres [62]. Nerome et al. (2013) [71] investigated the best operational condition to produce nanoparticles of lycopene/β-cyclodextrin using SEDS. The inclusion complex, which was prepared in N,N-dimethylformamide, was dissolved in the same solvent and then micronized by SEDS, using CO_2_ as a supercritical antisolvent. The effects of the initial concentrations of lycopene (0.35 mg/mL) and β-cyclodextrin (0.74 mg/mL), the CO_2_ flow rate (15, 20, 25 mL/min), the solution flow rate (0.25, 0.50, 0.75 mL/min), the pressure (10, 12, 14 MPa) and temperature (40, 45, 50 °C) at which the process was conducted were evaluated. The results obtained indicate that particles of the lycopene/β-cyclodextrin complex with morphologies from spherical to agglomerated were produced at the various conditions tested. The smallest particle size of 38 nm was obtained at high temperature and high pressure with a low solution flow rate, proving SEDS to be a promising technique in this study.

### 5.3. Stabilization of Phycobiliproteins

To the best of our knowledge, information regarding phycobiliproteins microencapsulation is scarce. Ganesan and Shanmugam (2020) [35] incorporated encapsulated phycoerythrin in ice creams. Phycoerythrin was extracted from the red seaweed *Kappaphycusalvarezii* with sodium–phosphate buffer and purified by ion-exchange chromatography. The purified pigment was encapsulated with kappa–carrageenan or guar gum to measure the stability and functionality in ice cream. Results showed that phycoerythrin exhibited dark pink color and has three distinct band subunits at α-20 kDa, β-21 kDa, and γ-30 kDa in SDS-PAGE. Its purity index was 2.32 (A563/A280 nm). The encapsulation efficiency (82.56%) and load percentage (56.78%) of carrageenan encapsulates were better than for guar gum encapsulates, while the opposite was obtained for hygroscopicity index and water solubility. Carragenan–phycoerythrin had particle sizes of 10–80 μm, while that of guar gum were larger (25–60 μm), showing that guar gum has more stickiness to the particles and aggregated on the pigment. In contrast, carrageenan as wall material showed intact appearance on phycoerythrin. Ice cream with added microencapsulated phycoerythrin showed better rheology, and the intensity of pink color increased during 90 days of storage. After the storage period, guar gum encapsulates show better stability and color in ice cream compared with carrageenan encapsulates. Concerning the antioxidant activity of the produced ice creams, samples with nonencapsulated phycoerythrin displayed better DPPH scavenging potential and FRAP reducing capacity than the encapsulated ones. However, after 90 days of storage, the opposite was verified.

## 6. Aquaculture Sustainable Production of Seaweed-Derived Pigments

The biosynthesis and accumulation of chlorophylls, carotenoids, and phycobiliproteins in seaweeds are strongly influenced by environmental factors such as nutrient availability, light intensity, and cultivation methods. The adoption of aquaculture practices for pigment production not only ensures a consistent biomass supply but also alleviates pressure on wild populations while contributing to environmental, economic, and health-related sustainability goals. Research across diverse systems—including Integrated Multi-Trophic Aquaculture (IMTA), land-based tanks, and sea-based farms—consistently highlights the importance of nutrient enrichment, particularly with nitrogen (commonly supplied as ammonium), in enhancing pigment synthesis and overall biomass quality [74,75,76,77,78]. For example, *Gracilaria chilensis* cultivated near salmon farms within an IMTA framework exhibited high photosynthetic efficiency and relative electron transport rates, which correlated with stable chlorophyll content and improved growth, thereby outperforming traditional bottom-culture methods [76].

Boderskov et al. (2016) [79] conducted an experiment with *Saccharina latissima* in outdoor tanks, during fall/early winter, exposed to ambient light and temperature variations. Air temperature and photosynthetically available radiation (PAR) gradually decreased from October to December. Despite a biomass reduction of 16.2–18.7%, high nutrient availability led to significant increases in the absolute harvestable amounts of nitrogen (by 50.1–60.1%), fucoxanthin (by 21.7–53.7%), and chlorophyll a (by 47.0–73.5%). On the other hand, under low nutrient availability, there was a net loss of biomass (8.1–9.5%), tissue nitrogen (10.7–44.1%), and fucoxanthin (7.1–17.2%), and a minor increase in chlorophyll a (2.5–22.8%). These findings underscore the importance of nutrient supply in balancing seasonal declines in photosynthetic pigments.

Rugiu et al. (2021) [77] evaluated how different nutrient concentrations affect growth, photosynthesis, chemical composition, and pigment content in *Saccharina latissima.* Three conditions were studied: (i) seaweeds in natural seawater (control); (ii) surface seawater enriched by pulses of ammonium nitrate (NH_4_NO_3_) mimicking the dissolved inorganic nitrogen concentration within the natural range of those produced by finfish cages (IMTA1); (iii) the nitrogen enrichment described for IMTA1 plus the water coming from the surface seawater enriched by blue mussel effluents (IMTA2). Compared to the control group, the photosynthetic responses and total nitrogen content were positively affected by both IMTA1 and IMTA2. For chlorophyll a, total carotenoids and fucoxanthin, the yield of pigments found in the algal tissue was highest for IMTA2 than for IMTA and control seawater, demonstrating that the highest pigment levels are associated with greater photosynthetic performance and nitrogen uptake.

In another study, Syamsuddin et al. (2019) [80] compared the growth, carotenoid, fiber and mineral content of *Caulerpa lentillifera* cultivated indoors and in the coastal waters. For indoor cultivation, two parameters were studied: (1) substrate composition—75% sand + 25% coral fragments; 50% sand + 50% coral fragments; 25% sand + 75% coral fragments; (2) seed spacing distance—5 cm, 10 cm, 15 cm, and 20 cm, on the mixed substrate of 50% sand and 50% coral fragments. For outdoor cultivation, the authors evaluated the effect of seed weight—50 g, 100 g and 150 g seeds/tray. Results showed that growth and mineral content were higher in specimens cultivated indoors, maybe due to the presence of readily absorbed minerals in the substrate provided, a mixture of sand and coral fragments. On the other hand, higher carotenoid and fiber content was potentiated by the cultivation in coastal waters probably because carotenoid synthesis occurred more intensively to protect chlorophyll from damage, leading to an increase in photosynthesis. Because of the photosynthesis intensification, the production of carbohydrates (fibers) was also improved.

Phycobiliproteins also demonstrated variable responses depending on nutrient availability and light conditions. Felaco et al. (2020) [75] observed that the phycobilin content in *Solieria filiformis* declined significantly in the absence of nutrient inputs (control group), regardless of light availability. However, when integrated with fish (treatment 1) and fish + sea cucumber (treatment 2) aquaculture, nutrient access was restored, leading to enhanced pigment (chlorophyll a and phycobilins) content and growth rates, and improved flexibility and morphology.

Stedt et al. (2022) [76] investigated the growth rates and crude protein content of four seaweed species—*Saccharina latissima*, *Ulva fenestrata*, *Ulva intestinalis*, and *Chaetomorpha linum*—cultivated in two ammonium concentrations (20 and 200 μM) derived from eight types of process water originating from recirculating salmon aquaculture systems, as well as from herring, shrimp, and oat processing. They concluded that the growth rates of the green seaweed were up to 64% higher, and crude protein quadrupled when cultivated in the food production process waters, compared to seawater controls. Growth rates were generally higher in the presence of 20 μM compared to 200 μM ammonium, while crude protein content was either unaffected or positively affected by the increasing ammonium concentration. Additionally, the color of the seaweed thallus was maintained or darkened with the food process waters, the latter possibly indicating higher chlorophyll concentration. These results suggest that nutrient recycling from industrial or aquaculture effluents can significantly boost pigment-related metabolic pathways.

Based on this data, we can conclude that pigment production in seaweeds is strongly influenced by environmental nutrient levels, particularly nitrogen, and is modulated by cultivation method and light exposure. Optimizing these parameters in aquaculture systems not only boosts biomass productivity but also improves the biochemical composition of the harvested seaweed, increasing their nutritional and commercial value for use as food additives.

## 7. Current Legal Framework

Table 3 provides an overview of the current legal framework governing seaweed-derived pigments (chlorophylls, carotenoids, and phycobiliproteins) in the European Union and the United States.

### 7.1. Chlorophylls as Food Additive

In Europe, chlorophylls and chlorophyllins are approved under E140 (chlorophylls) and E141 (copper complexes of chlorophylls and chlorophyllins) [90]. Maximum permitted levels vary by food category and are listed in Regulation (EU) No 1129/2011 [82]. They are used as coloring agents in several foods, namely, confectionery, ice cream and desserts, beverages, sauces, and dressings.

In the United States, chlorophyll additives are regulated and authorized by the Food and Drug Administration (FDA) under its general framework for the use of substances as color additives, specified in the Code of Federal Regulations (CFRs), Parts 21 CFR 73, 74, or 82 [83,84]. The FDA exercises oversight via food additive petitions and the GRAS (Generally Recognized as Safe) notification program. Sodium copper chlorophyllin is approved for use in food preparations such as citrus-based dry beverage mixes at a maximum concentration of 0.2% in the dry mix (21 CFR 73.125). Regarding production, well-defined specifications must be met for sodium copper chlorophyllin to be used as a food colorant. The raw material for its extraction is restricted to alfalfa, and the extraction methodology must be clearly defined, including information on the solvents employed, which may include acetone, ethanol, hexane, or combinations thereof. The process includes preparation of the water-soluble component by saponification and substitution of the magnesium atom naturally present in chlorophyll with copper. Safety standards must also be observed; for example, the amount of free copper should not exceed 200 parts per million (ppm), while total copper content must range between 4 and 6%. Moreover, limits are set for lead (≤10 ppm), arsenic (≤3 ppm), mercury (≤0.5 ppm), and residual solvents, which must not exceed 50 ppm. Certification of this color additive is not required for the protection of public health, and thus, batches are exempt from certification requirements, in contrast to many synthetic color additives [83,84].

### 7.2. Carotenoids as Food Additive

In Europe, chlorophylls and chlorophyllins are approved under E140 (chlorophylls) andaccording to EU legislation [81,82], carotenoids appear as the E160 series. In this series the mixed carotenes and β carotenes having plant/seaweed origin are identified as E160a. All E160 additives are colorants, authorized across a broad range of food categories, such as beverages (e.g., juices, soft drinks), confectionery and fine bakery wares, dairy products, spreads, dressings, sauces, snack foods, soups, canned, processed fruits/vegetables, processed foods, and ice creams. β carotene (E160a(ii)) is additionally permitted in foods for special medical purposes [86]. The regulation 231/2012 [91] establishes specifications for food additives and defines crucial purity criteria and technical specifications that manufacturers must meet for carotenoid additives. For extracts, the content of the main carotenoid (e.g., β carotene, lutein, lycopene, etc.) must meet minimum assay values. For E160a(i), a β carotene from *Blakesleatrispora*, the content should be ≥10% of the total carotenoids and for E160a(ii) (synthetic all-trans β carotene): ≥96% all-trans β carotene. Also, the regulation defines maximum limits for residual solvents (ethanol and others, depending on the process), for the presence of impurities (like other isomers or related carotenoids), for heavy metals (lead, arsenic, cadmium and mercury), for ethylene oxide, and for residual traces of pesticides. For ethylene oxide, the total limit (sum of ethylene oxide +2-chloro-ethanol, expressed as ethylene oxide) is 0.1mg/kg. The maximum levels for heavy metal levels are lead: ≤1mg/k; cadmium: ≤1mg/kg; arsenic: ≤1mg/kg; and mercury: ≤0.1mg/kg. The isomeric impurities (e.g., cis isomers) must be within varying assay thresholds, typically β carotene: ≥96% alltrans; and mixed sources: ≥10% total carotenoids. Other related carotenoids and by-products are limited by HPLC purity criteria specified per E-number. Permitted solvents vary by carotenoid and extraction process. Solvents usually allowed for extraction are ethyl acetate, acetone, carbon dioxide, dichloromethane, n-butanol, methanol, ethanol, hexane, and 2-propanol. Residual solvent limits follow the International Council for Harmonization(ICH) and the Joint FAO/WHO Expert Committee on Food Additives (JECFA) guidelines; typically, ≤0.5% *w*/*w* of final product [92]. Residual traces of pesticides are allowed but must not exceed levels found in starting plant or microbial materials and must meet GC/MS detection at trace levels. If residues exceed 0.01 mg/kg, a risk assessment per Regulation (EC) No 396/2005 is required [93]. For mixed carotenes (E160a (i)) and β-carotene (E160a (ii)), the European authority could not establish an Acceptable Daily Intake (ADI) based on available data, concluding that their use as food color is not a safety concern provided that the intake remains ≤15 mg/day from all sources [85].

In the USA, carotenoid food additives are regulated under a dual-framework system administered by FDA. Depending on their intended use as color additives or as nutritional supplement, carotenoids fall under different parts of title 21 of the Code of Federal Regulations (CFRs). Carotenoids used as colorants are regulated under 21 CFR Part 73 (Color Additives Exempt from Certification) [94], while those used as nutritional ingredients or food additives are governed under 21 CFR Part 170 (Food Additives including GRAS provisions) [95]. This dual classification allows carotenoids to function both as coloring agents and as nutrient supplements in various food applications. β-Carotene may be used as color additive (21 CFR 73.95) [87] and in this case, it is exempt from certification and authorized for general use in foods consistent with good manufacturing practice. This carotenoid is also allowed to act as a nutrient additive because it is a substance Generally Recognized as Safe (GRAS) under 21 CFR 184.1245 [88]. β-carotene can be used in dietary supplements and fortified foods. According to US legislation (21 CFR 70.25) [96], carotenoids as color additives must be labeled as “color added,” “artificial color,” or “artificial color added.” Certified colorants must bear specific names, while exempt color additives may use generic terms unless otherwise required.

### 7.3. Phycobiliproteins as Food Additives

Phycobiliproteins, notably phycocyanin and phycoerythrin, are increasingly explored as natural food colorants [97,98]. The legal status of phycobiliproteins as food additives is evolving, with phycocyanin from spirulina extract gaining some approvals, particularly in the USA. However, both phycocyanin and phycoerythrin face regulatory hurdles in regions like the EU, where comprehensive safety assessments are required [99].

Phycocyanin has not been approved as a food colorant in the EU [82,86]. This is due to the high costs of compliance with safety regulations and lack of standardized guidelines hindering small enterprises’ participation, as well as the lack of harmonizing national interpretations of regulations, even though seaweed aligns with the EU’s Green Deal and farm-to-fork strategy for low-carbon food systems [6]. In the USA, phycocyanin (from spirulina extract) is approved as a color additive exempt from certification and listed under 21 CFR § 73.530 [89]. It is used as coloring agent in a variety of food products, such as candies, chewing gum, ice cream, frozen desserts, and (non-alcoholic) beverages. Similar to what happens in the USA, phycoerythrin has not yet received approval as a food additive in the EU, and its acceptance would require substantial scientific evidence.

## 8. Limitations and Perspectives of Seaweed-Derived Pigments Applications

Despite all the examples provided in the previous sections on the application of seaweed pigments for food product development currently, the food industry still rely more on plant-based pigments (such as anthocyanins, carotenoids, and betalains), which are abundant in widely cultivated crops (fruits, vegetables, roots) and can be extracted in large amounts from raw materials and food by-products. These pigments are less costly than seaweed-derived pigments, which require aquaculture systems or wild harvesting and are subject to seasonal, geographic, and environmental variability [100]. Moreover, plant pigments are already widely approved as food colorants in different countries, whereas seaweed extracts may contain heavy metals and iodine above permitted limits, creating the need for additional toxicological validation before large-scale approval. Another factor relates to consumer acceptance: consumers are more familiar with plant-based pigments, often associated with fruits and vegetables and perceived as “natural and healthy,” while seaweed pigments are less well known, and their oceanic origin can sometimes raise doubts about taste, safety, or contamination risks [100]. There are several studies reporting on the incorporation of plant-based pigments in food products, such as, the fortification of traditional tapioca “pancakes” with microencapsulated carrot carotenoids [101], bread enriched with microencapsulated anthocyanin extracts from various plant materials (cornelian cherry, red cabbage, chokeberry) [102], ice creams enriched with microencapsulated anthocyanins from black carrot [103], mayonnaise with sea buckthorn carotenoids [104], or an oat meal paste incorporating microencapsulated a carotenoid-rich extract from guaraná peels [105].

Nonetheless, interesting new avenues can be foreseen for seaweed pigments. The combination of chromaticity and functionality of seaweed pigments make them high-value compounds in next-generation food systems, such as incorporation in inks for 3D-foods, functional/smart packaging, AI-assisted nutrition design, and symbiotic concepts that blend pigments with probiotics and other bioactive compounds. Three-dimensional food printing already uses hydrocolloids and protein/starch matrices (“inks”) to control flow, layering, and post-print setting; these inks are proven vehicles for incorporating bioactives (vitamins, antioxidants, probiotics) while preserving sensory quality and antioxidant capacity after printing and post-treatments. Seaweed-derived ingredients naturally align with this: alginates/carrageenans can structure the ink; and pigments can deliver both natural colors and functionality [106,107]. Seaweed biopolymers can also be used to produce intelligent packaging. Incorporating seaweed pigments into these films can scavenge radicals and absorb UV, extending product shelf life, serving as natural pH indicators, and differentiating clean-label packages visually without synthetic dyes [108,109].

Combining seaweeds pigments and hydrocolloids with artificial intelligence and machine learning can also shorten the path from concept to robust product development allowing for faster formulation, stability prediction, and personalization, for instance, in the case of personalized 3D-printed functional foods [106,110]. Seaweed biomass supplies not only pigments but also prebiotic sulfated polysaccharides (e.g., fucoidan, ulvan, laminarin, alginate) that support beneficial gut bacteria [111] or anti-inflammatory compounds [112]. Combining seaweed prebiotics or anti-inflammatory compounds with naturally occurring seaweed pigments can create functional food ingredients that offer synergistic health benefits, including enhanced gut health and a rich source of antioxidants.

## 9. Concluding Remarks

Seaweed-derived pigments, chlorophylls, carotenoids, and phycobiliproteins, present a sustainable and multifunctional alternative to synthetic food colorants, offering both vibrant hues and health-promoting properties. Although recent advances in aquaculture, green extraction technologies, and encapsulation strategies have significantly improved the feasibility of pigment production and stability, challenges remain in scaling up these technologies, ensuring cost-efficiency, and aligning with food safety regulations. The present research remains limited, particularly regarding their stability, colorant functionality, toxicological safety, and efficient extraction and purification protocols. A comprehensive evaluation of these factors is essential to support the development of functional ingredients suitable for food applications.

One of the major research gaps lies in understanding how food processing conditions, such as thermal treatment, pH changes, and mechanical stress, affect the digestibility, bioavailability, and bioactivity of seaweed-derived pigments when incorporated into diverse food matrices. These parameters are critical for predicting the functional efficacy of pigments like chlorophylls, carotenoids, and phycobiliproteins in real-world applications.

From a technological perspective, the large-scale production of seaweed-derived pigments remains a considerable challenge. This is primarily due to the complexity, low efficiency, and high costs associated with existing extraction and purification processes. The recovery of seaweed-derived pigments often involves multiple steps, including cell disruption, aqueous extraction, precipitation, and chromatographic purification. These procedures are not only time- and labor-intensive, but also susceptible to losses in pigment quality and yield due to the photo- and thermo-sensitivity of natural pigments. Although some progress has been made, the economic feasibility and scalability of these approaches remain unproven at the industrial level. Moreover, the volatility, low extraction yield, and market price sensitivity of seaweed pigments further limit their commercial viability.

The instability of seaweed-derived pigments, especially during processing and storage, poses another significant barrier to their widespread application. For example, chlorophyll, a labile magnesium porphyrin compound, readily degrades in the presence of acids, bases, light, and oxidizing agents. Recent studies have investigated the use of nanoencapsulation and microencapsulation techniques to improve pigment stability. Encapsulation materials such as sodium caseinate, as well as metal ion substitutions (e.g., replacing magnesium with copper or zinc), have shown promise in enhancing the structural integrity and application range of seaweed pigments.

Moreover, the current legal framework, particularly in the European Union and the United States, reflects a cautious yet evolving stance, with specific pigments receiving approval while others await comprehensive evaluation. For broader commercial adoption, further interdisciplinary efforts are needed to address regulatory gaps, validate safety and efficacy, and standardize production processes. Integrating innovation with clear legal pathways will be key to unlocking the full potential of seaweed pigments in the food industry.

## Figures and Tables

**Table 1:** Stability of seaweed-derived pigments in different food matrices.

Pigment Class	Food Matrix	Major Instability Factors	Molecular Interactions	Stabilization Strategies	Ref.
**Chlorophylls**	Acidic beverages, fruit juices	Acid-induced Mg^2+^ loss; light/heat degradation	Proton substitution destabilizes porphyrin; weak protection from proteins/lipids	pH adjustment; use of chlorophyllin; encapsulation	[53]
**Carotenoids** (fucoxanthin, β-carotene, astaxanthin)	Dairy emulsions, dressings, bakery products	Heat- and light-induced isomerization; oxidative cleavage	Partition into lipid droplets; binding to hydrophobic protein sites reduces oxidation	Nano and microencapsulation (gum arabic, maltodextrin)	[54]
**Phycobiliproteins** (phycocyanin, phycoerythrin)	Beverages, gels, confectionery	Denaturation at pH < 5; thermal unfolding (>55 °C); photo-oxidation	Electrostatic interactions with proteins and polysaccharides can partially stabilized	Encapsulation in alginate/pectin beads; coacervation with proteins	[57]

**Table 2:** Microencapsulation techniques employed to protect seaweed-derived pigments.

Pigments	Source	Stabilization Method	Encapsulation Agents	Food Product	Ref
Chlorophyll c, *trans*-fucoxanthin*cis*-fucoxanthin, zeaxanthin, pheophytin a, *E*-carotene	*Sargassum* sp.	Microencapsulation by freezedrying	Maltodextrin, tween–80	-	[65]
Chlorophylls a and b	*Caulerpa* sp.	Microencapsulation by freezedrying	Gelatin, gum Arabic, tween-80	Jelly drinks	[17]
Chlorophylls a, b, c, d	*Ulva intestinalis*	Microencapsulation by freezedrying and by spray drying	Maltodextrin and whey protein isolate	-	[66]
β-Carotene	Sigma–Aldrich (Burlington, MA, USA)	Microencapsulation by supercritical micronization (SEDS)	Copolymer poly(3-hydroxybutirate-co-hydroxyvalerate)	-	[67]
Lutein	Shanghai Winherb Medical Co., Ltd. (Shanghai, China)	Proliposomes by supercritical micronization (SAS 9.4)	Hydrogenated soya phosphatidylcholine		[68]
β-Carotene	Vitatene SA (Leon, Spain)	Microencapsulation by supercritical micronization (PGSS drying)	Poly-(ε-caprolactones)	-	[69]
β-Carotene	Vitatene SA	Microencapsulation by supercritical micronization (PGSS drying)	Soybean lecithin	-	[70]
Lycopene	Wako (Monza, Italy)	Microencapsulation by supercritical micronization (SEDS)	*β*-cyclodextrin	-	[71]
β-Carotene	Sigma-Aldrich	Proliposomes by spray drying	Phospholipon 90H and sucrose for liposome formation; liposomes stabilized with xanthan gum (thickening agent)	**-**	[72]
β-Carotene	MP Biomedicals (Irvine, CA, USA)	Microencapsulation by spray drying. Microspheres with chitosan–alginate beads	Maltodextrin; Chitosan; alginate	Pudding and yogurt	[27]
β-Carotene	Sigma-Aldrich	Microencapsulation by complex coacervation	Amaranth carboxymethyl starch and lactoferrin	Gummy candies	[28]
Fucoxanthin	*Sargassum thunbergii*	Microencapsulation by complex coacervation	Palm stearin, fish gelatin–gum arabic complex	-	[73]
Fucoxanthin	*Undaria pinnatifida*	Microencapsulation by spray drying	Different materials were tested: Hydroxypropyl-β-cyclodextrin, maltodextrin, gum arabic, whey protein isolate, isolated pea protein, and gelatin	-	[59]
Phycoerythrin	*Kappaphycusalvarezii*	Microencapsulation by freezedrying	Kappa–carrageenan and guar gum	Ice cream	[35]

**Table 3:** Legal framework of seaweed-derived pigments.

	Europe Union	United States (FDA)
**Chlorophylls**		
Regulation	Regulation (EC) No 1333/2008 [81,82]	CFR 73.125 [83,84]
Additive code	E140 (chlorophylls); E141 (copper complexes of chlorophylls and chlorophyllins)	Chlorophyllin copper complex
Status	Approved as colorants	Limited approved uses
Uses	Confectionery, ice cream and dessert, beverages, sauces and dressings	Citrus-based dry beverage mixes
Permitted levels	Regulation (EU) No 1129/2011 according to food categories [82]	Maximum concentration of 0.2% in the dry mix
**Carotenoids**		
Regulation	Regulation (EC) No 1333/2008 [85,86]	21 CFR Part 73 and GRAS [87,88]
Additive code	E160a–f, E161b–g	
Status	Approved as colorants	Permitted carotenoids: β-Carotene, Lutein
Uses	Margarine, cheese, snacks, beverages and desserts, bakery and confectionery	
Permitted levels	Regulation (EU) No 1129/2011according to food categories [82]	
**Phycobiliproteins**		
Regulation	Regulation (EC) No 1333/2008 [86] Regulation EU No 1129/2011 [82]	CFR § 73.530 (Spirulina extract) [89]
Additive code	Not approved as additive	
Status	Allowed as coloring foodstuff	Approved (Spirulina extract)
Uses	Beverages, desserts, candies, chewing gum, ice cream	Beverages, desserts, candies, chewing gum, ice cream

Food and Drug Administration (FDA), Generally Recognized as Safe (GRAS).

## Data Availability

No new data were created or analyzed in this study. Data sharing is not applicable to this article.
